# *Campylobacter jejuni* permeabilizes the host cell membrane by short chain lysophosphatidylethanolamines

**DOI:** 10.1080/19490976.2022.2091371

**Published:** 2022-07-07

**Authors:** Xuefeng Cao, Chris H.A. van de Lest, Liane Z.X. Huang, Jos P.M. van Putten, Marc M.S.M. Wösten

**Affiliations:** Department Biomolecular Health Sciences, Utrecht University, Utrecht, Netherlands

**Keywords:** Lysophospholipids, lysophosphatidylethanolamines, novel virulence factor, *Campylobacter jejuni*, cytotoxicity, hemolysis

## Abstract

Lysophospholipids (LPLs) are crucial for regulating epithelial integrity and homeostasis in eukaryotes, however the effects of LPLs produced by bacteria on host cells is largely unknown. The membrane of the human bacterial pathogen *Campylobacter jejuni* is rich in LPLs. Although *C. jejuni* possesses several virulence factors, it lacks traditional virulence factors like type III secretion systems, present in most enteropathogens. Here, we provide evidence that membrane lipids lysophosphatidylethanolamines (lysoPEs) of *C. jejuni* are able to lyse erythrocytes and are toxic for HeLa and Caco-2 cells. Lactate dehydrogenase (LDH) release assays and confocal microscopy revealed that lysoPE permeabilizes the cells. LysoPE toxicity was partially rescued by oxidative stress inhibitors, indicating that intracellular reactive oxygen species may contribute to the cell damage. Our results show that especially the short-chain lysoPEs (C:14) which is abundantly present in the *C. jejuni* membrane may be considered as a novel virulence factor.

## Introduction

Lysophospholipids (LPLs) are bioactive signaling molecules containing a single fatty acid tail. In eukaryotic cells, LPLs exhibit diverse biological properties, such as promoting cell growth, acting as potent lipid mediators, or reducing bacterial infections.^1,2^ LPLs are generated as metabolic intermediates in phospholipid synthesis or during membrane degradation.^3^ The formation of LPLs from phospholipids is due to activation of phospholipase A1 or A2. Phospholipase A1 (PldA_1_) and phospholipase A2 (PldA_2_), hydrolyzing the stereospecific numbering (*Sn*)-1 and −2 acyl chain, respectively.^4^ (*Sn*)-1 LPLs possess more shorter, saturated acyl chains than (*Sn*)-2 LPLs while (*Sn*)-2 LPLs possess more unsaturated acyl chains.^5^ (*Sn*)-1 LPLs and (*Sn*)-2 LPLs might have different biological functions as only (*Sn*)-1 LPLs can act as mediators of antimicrobial activity toward Gram-positive bacteria.^6^ Lysophosphatidic acid (lysoPA) is important in controlling and signaling cancer;^7^ lysophosphatidylcholine (lysoPC) evokes cellular injury by oxidative events that involve formation of low-density lipoprotein. Both lysoPA and lysoPC of the host trigger the release of the proinflammatory flagellin from *Salmonella* thereby enhancing the innate and inflammatory responses toward this bacterium.^8^ The role of other LPLs like lysophosphatidylethanolamine (lysoPE) has not been elucidated to such a high degree.

Bacteria usually contain small amounts (<1%) of LPLs in their membrane,^3^ mostly found in the form of lysoPE.^9^ It has been mentioned that lysoPEs isolated from *Bacteroidetes Chitinophaga* spp. have antimicrobial activities against certain Gram-positive bacteria.^10^ LysoPA and its precursor lysoPC derived from *Lactobacillus plantarum* has been considered being toxic for humans and could disturb the signaling networks in host cells.^11^ The biological function of LPLs in bacteria is still poorly understood, but they may play a role in bacterial survival or invasion.^2^ LPLs may be an underestimated factor in bacterial pathogenesis and inflammation response of the host.

We previously showed that the bacterial pathogen *Campylobacter jejuni* possesses a wide spectrum of LPLs that varies dependent on the environmental conditions.^12^ *C. jejuni* is the leading cause of bacterial foodborne human gastroenteritis in developed countries.^13^ Symptomatic infection typically involves intestinal inflammation, fever, and bloody diarrhea.^13^ *C. jejuni* is supposed to penetrate the intestinal mucus layer, colonize the crypts, and disrupt the epithelial barrier.^14^ Although *C. jejuni* possesses a number of virulence factors, such as flagella, proteases, adhesins, type VI secretion system, and cytolethal distending toxin, it lacks traditional virulence factors like type III secretion systems,^15^ present in most enteropathogens and therefore the molecular basis of *C. jejuni* infection is still poorly understood.^16^

In the present study, we investigated the biological role(s) of the LPLs of *C. jejuni* as potential virulence determinant. We demonstrate that *C. jejuni* PldA generates both (*Sn*)-1 and (*Sn*)-2 LPLs. The generated short-chain fatty acids lysoPE was found to exert hemolytic activity and effectively damage different types of eukaryotic cells, indicating that it may act as a virulence factor.

## Results

### C. jejuni *PldA produces (*Sn*)-1 as well as (*Sn*)-2 LPLs*

**Figure 1.**
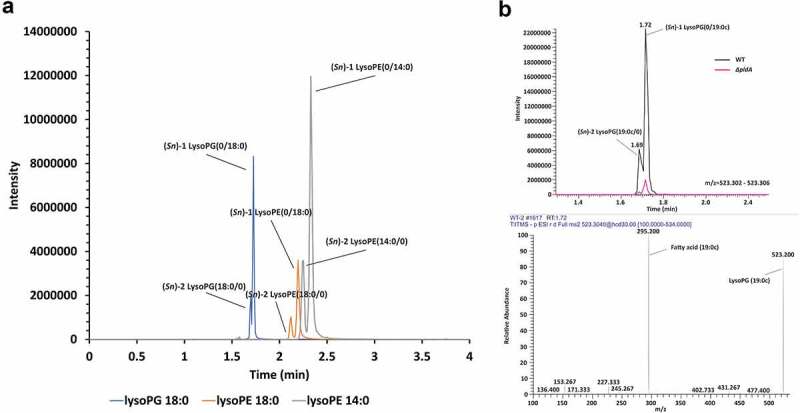
**Phospholipid cleavage by *C. jejuni* PldA**. LC-MS spectra of (a) the main LPLs in wildtype *C. jejuni* strain 81116. (b) lysoPG (19:0c) in *C. jejuni* wildtype and *C. jejuni ΔpldA.*

To ensure that *C. jejuni* produced lysolipids under the conditions employed, we extracted LPLs from 16 h old cultures of *C. jejuni* wildtype 81116 and its isogenic Δ*pldA* mutant strain growth under microaerophilic conditions at 42°C. Liquid chromatography tandem mass spectrometry (LC-MS/MS) of extracted lipids clearly demonstrated the presence of both (*Sn*)-1 and −2 acyl chain LPLs in wildtype *C. jejuni*. For example, the majority of lysoPG 18:0 was present as (*Sn*)-1 lysoPG (0/18:0), while (*Sn*)-2 lysoPG (18:0/0) was roughly 4 times less detected (Figure 1a). Both lysoPG species were virtually absent in a mutant strain lacking a functional PldA (Figure 1b). Similar results were observed for other LPLs. This indicates that PldA is the primary enzyme involved in LPL formation in *C. jejuni* and that this enzyme is able to cleave both (*Sn*)-1 and −2 acyl chains but prefers the (*Sn*)-1 site.

### C. jejuni *LPLs cause erythrocytes to lyse*

**Figure 2.**
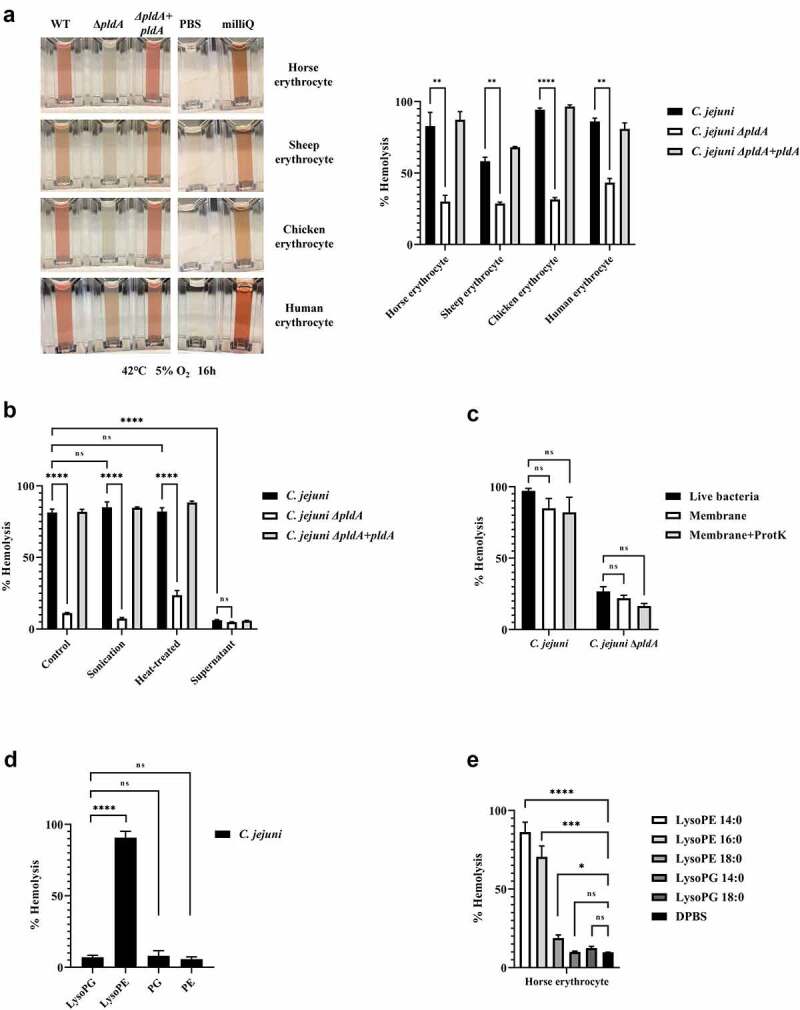
***C. jejuni* lysoPE induced hemolysis**. (a) *C. jejuni* strains were incubated with erythrocytes from different species; the hemolysis results depicted in the left panel were quantified by measuring absorbance at 420 nm. (b-e) Hemolysis of horse erythrocytes after incubation with: (b) live, heat-treated or sonicated *C. jejuni*, or with the cell-free supernatant of *C. jejuni*; (c) live bacteria, whole membrane or protease K-treated membranes of *C. jejuni*; (d) purified major phospholipid classes; (e) commercially available LPLs. MilliQ water and DPBS were used as positive (100% value) and negative (0% value) control in the hemolysis assay, respectively. Data of three independent experiments with three independent preparations of bacterial samples are presented as mean values ± standard deviation, *P < .1, **P < .01, ***P < .001, ****P < .0001, ns P > .1.

To test the effect of bacterial LPLs on eukaryotic cells, we first determined the hemolytic activity of wildtype *C. jejuni* and the *ΔpldA* mutant. Wildtype *C. jejuni* caused strong hemolysis of horse erythrocytes in contrast to *C. jejuni ΔpldA*. Complementation of the mutant (*C. jejuni ΔpldA +pldA*) restored the strong hemolytic activity (Figure 2a). No hemolysis was observed by using *C. jejuni* cell-free culture supernatant (Figure 2b) nor when the pellet fraction of ultracentrifugated cell-free culture supernatant was used, suggesting that the hemolysis required bacteria-host cell contact. In other bacterial species phospholipase A itself has been shown to induce hemolysis. These enzymes generally prefer phosphatidylcholine (PC) as substrates.^17^ *C. jejuni*-mediated hemolysis was observed for both PC-rich (horse, chicken and human) and PC-deficient (sheep) cells indicating that the hemolysis was PC independent (Figure 2a). Furthermore, the *C. jejuni*-induced hemolysis was maintained after heating of the bacteria (75°C, 30 min) (Figure 2b), indicating that the activity was insensitive to denaturation. Hemolysis was also still present when horse erythrocytes were incubated with isolated membranes of *C. jejuni*, even after proteinase K treatment followed by heat inactivation (Figure 2c). These results together strongly suggest that *C. jejuni* LPLs are causing hemolysis.

### Short lysoPEs are responsible for hemolysis

In order to determine the phospholipid species responsible for the red blood cell lysis, we separated the major phospholipid classes PG, PE, lysoPG, and lysoPE from *C. jejuni* wildtype. The purity of the phospholipid classes samples are shown figure S1. Minimal lysis was observed when PG, PE, or lysoPG were incubated with horse erythrocytes, whereas the lysoPE fraction lysed more than 90% of the cells (Figure 2d). This indicates that *C. jejuni* lysoPE is the primary cause for hemolysis. Next, we investigated the effect of the length of the fatty acid tail using commercial lysoPE species. All lysoPE species induced hemolysis but the shortest fatty acid tail containing lysoPE were most effective (Figure 2e). Of note, lysoPE14 and lysoPE16 make up 50% of the *C. jejuni* lysoPE molecules.^12^ Together, these results indicate that short lysoPE species disrupt the integrity of the cell membrane of horse erythrocytes.

### Short lysoPEs are also toxic for epithelial cells

**Figure 3.**
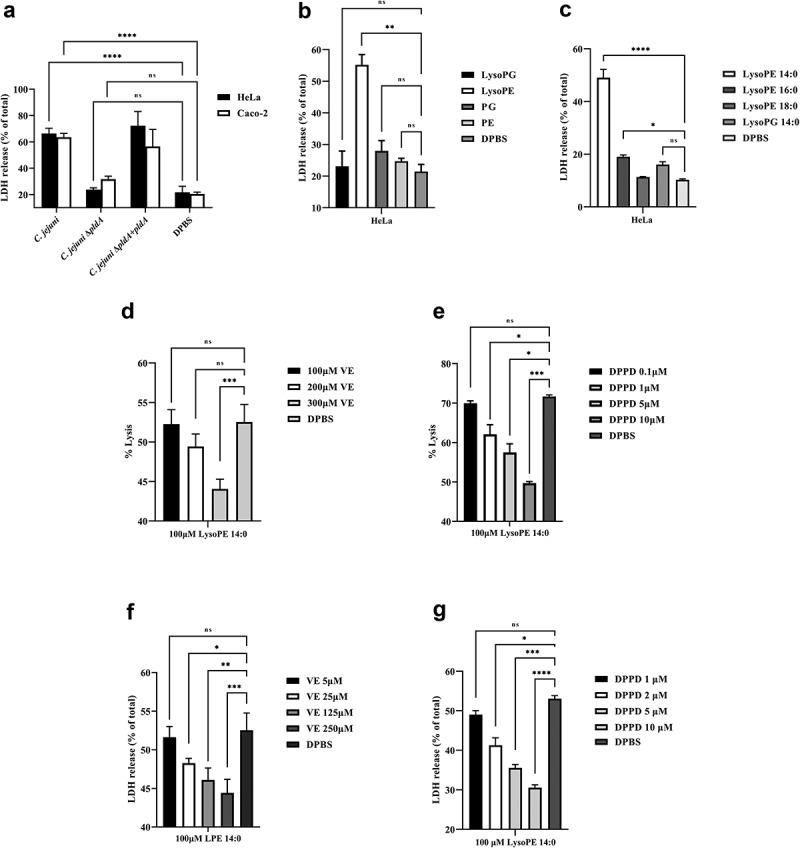
***C. jejuni* lysoPE-induced toxicity for host cells rescued by vitamin E and DPPD**. (a) LDH release of HeLa and Caco-2 cells treated with *C. jejuni* strains . (b) HeLa cells treated with purified phospholipid fractions. (c) HeLa cells treated with commercially available LPLs. (d-e) Horse erythrocytes and (f- g) HeLa cells were treated without or with vitamin E or DPPD, washed and then exposed to lysoPE 14:0. Data are from three independent experiments with three independent preparations of bacterial samples and presented as mean values ± standard deviation, *P < .1, **P < .01, ***P < .001, ****P < .0001, ns P > .1.

During the natural infection, *C. jejuni* is in close contact with mucosal epithelial cells. To determine whether lysoPE may also damage epithelial cells, we measured the LDH release. Wildtype *C. jejuni* caused considerable LDH release from the human HeLa and Caco-2 cells after 5 h of incubation. This effect was much less for *ΔpldA* mutant, while the complemented *C. jejuni ΔpldA +pldA* mutant regained the harmful wildtype behavior (Figure 3a). Strong LDH release was also observed after exposure to purified *C. jejuni*-derived lysoPE (Figure 3b). In agreement with the hemolysis, the short fatty acid tail containing lysoPE 14:0 caused the highest LDH release (Figure 3c). Together, the results indicate that short-chain lysoPE as present in *C. jejuni* not only displays hemolytic activity but also causes damage to epithelial cells.

### LysoPE induces cell damage by oxidative stress

**Figure 4.**
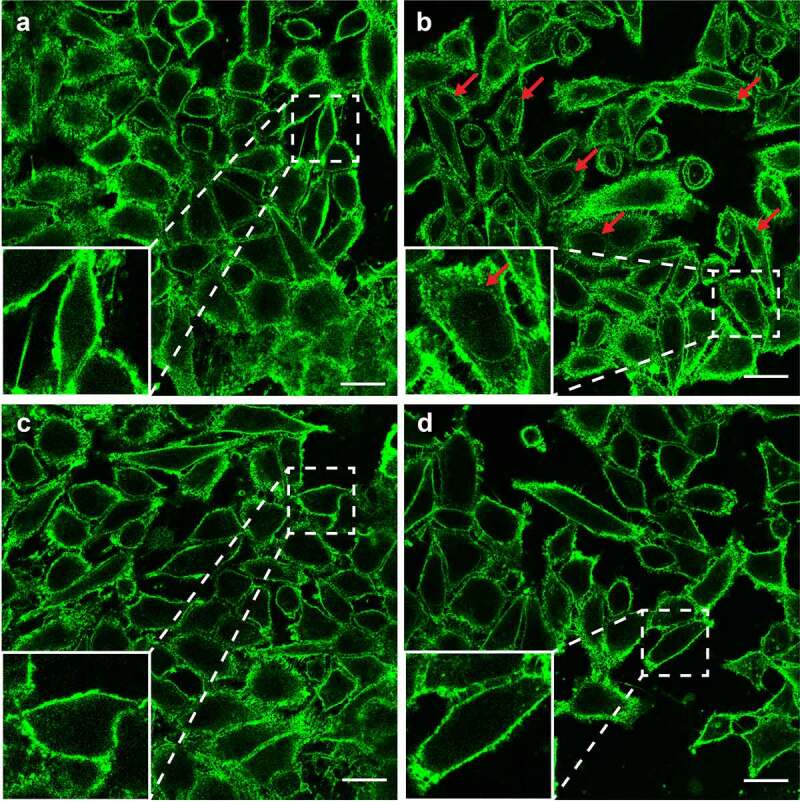
**LysoPE permeabilizes the epithelial cells membrane**. HeLa cells incubated with (a) DPBS, (b) lysoPE 14:0, (c) lysoPG 14:0, or (d) pre-treated with vitamin E, washed and incubated with lysoPE 14:0 were stained with the membrane stain fluorescent WGA (green) and/or nuclear DAPI stain (blue) and visualized by confocal microscopy. Red arrows point to nuclear membrane stained with WGA. White scale bars represent 5 μm.

To investigate whether the lysoPE-induced cell damage is due to oxidative stress as seen for lysoPC,^18^ the effect of two antioxidants, vitamin E, and DPPD, was examined. Horse erythrocytes were pre-exposed to antioxidant, and then incubated with lysoPE 14:0. For both antioxidants, a clear concentration-dependent inhibition of lysoPE-induced hemolysis was observed (Figure 3d,e). Experiments with epithelial cells yielded similar results with significantly less damage after pre-treatment of cells with vitamin E and DPPD (Figure 3f,g). To corroborate these findings we applied confocal microscopy. In the absence of LPLs, epithelial cell membranes were impermeable to the green fluorescent lectin WGA (Figure 4a & S2A). However, after incubation with lysoPE 14:0 also nuclear membranes became WGA-positive (red arrows in Figure 4b & S2B), but not for lysoPG 14:0 treatment (Figure 4c & S2C), indicating that lysoPE enabled the lectin to pass the plasma membrane and enter the cells. Pretreatment of the cells with antioxidant prior to lysoPE 14:0 treatment prevented nuclear membrane staining (Figure 4d & S2D) consistent with the LDH release results. Together, these results point to oxidative stress as a major factor in the short chain lysoPE-induced cell damage.

## Discussion

In eukaryotic cells, LPLs play an essential role in a broad variety of biological processes.^1^ Recently, the human gut microbiota has been shown to contribute to the production of lysoPC, which causes damage of the epithelial barrier.^19^ Some bacterial pathogens, including *C. jejuni*, can produce large amounts of LPLs but their effect on host cell biology is largely unknown. Here, we show that the PldA of *C. jejuni* possesses phospholipase activity that generates (*Sn*)-1 and (*Sn)*-2 LPLs which is more typical for phospholipase class B proteins.^20^ We also for the first time provide evidence that the produced lysoPE phospholipids can lyse erythrocytes and damage epithelial cells. This effect is especially evident for short-chain lysoPE species and can be rescued by oxidative stress inhibitors. These results indicate that *C. jejuni* lysoPE may be an important unforeseen bacterial virulence factor that causes cell damage (at least partially) via an oxidative stress-sensitive mechanism.

The finding that *C. jejuni* PldA generates both (*Sn*)-1 and (*Sn*)-2 LPLs was unexpected as the amino acid sequence characteristics suggest that the enzyme belongs to the phospholipid class A family of proteins.^21^ The observed virtual absence of LPLs in *C. jejuni ΔpldA* indicates that no other phospholipases are active. However, our LC-MS/MS results clearly indicate that the *C. jejuni* PldA enzyme prefers to cleave at the (*Sn*)-1 site. The position of (*Sn*) cleavage is relevant as PldA_1_ generates mostly saturated LPL, while PldA_2_ generates mostly unsaturated or cyclo phospholipids,^5^ with different biological effects on membrane function.^22^ The finding that the *C. jejuni* PldA enzyme prefers to cleave at the (*Sn*)-1 site implies the formation of a large amount of membrane integrity reducing LPLs.^22^

The first evidence of a cytotoxic effect of *C. jejuni* LPLs was the observed hemolysis caused by *C. jejuni* wild type but not *C. jejuni ΔpldA*. Complementation of the *pldA* defect confirmed the crucial role of LPL formation in the toxicity. *C. jejuni*-induced hemolytic activity has previously been reported for both type VI secretion system-positive and negative *C. jejuni* strains, but the causing factor is still unclear.^23,24^ It has been speculated that the hemolysis was due to an intracellular component released after cell death or lysis,^25^ or by the PldA directly targeting host cells membranes. Here we provide evidence that the PldA products, the LPLs, exert strong hemolytic activity.

Fractionation of the major phospholipid classes of *C. jejuni* identified lysoPE as prime hemolysis inducing factor (Figure 2d). So far only lysoPA and lysoPC have been reported to affect erythrocytes.^26^ We found that besides the head group, also the length of the tail of the lysoPE is important for hemolysis as especially short lysoPEs were toxic (Figure 2e). This resembles observations with lysoPC where increasing the chain length of the hydrophobic tail decreases the rate of the hemolytic reaction.^27^ According to our previous results the phospholipidome of *C. jejuni* can consists of more than 33% lysoPE of which almost 50% is present as lysoPE 14 and 16.^12^ This likely explains why the membranes of live or dead *C. jejuni* bacteria are toxic for erythrocytes.

Interestingly, the cytotoxicity of *C. jejuni* membranes and purified lysoPE was also observed for epithelial cells as evident from the strong PldA dependent increase of LDH release and the staining of intracellular membranes with WGA in lysoPE-treated cells only (Figure 4b & S2B). Maximum LDH release and intracellular staining were observed after exposure to short chain fatty acid containing lysoPE (Figure 3c). In humans, short-chain fatty acids have been identified as signaling molecules between the gut microbiota and the host, and are regarded as toxic at high concentration.^28^ *C. jejuni* has been shown to induce LDH release in human neutrophils and dendritic cells (less than 10%), but in epithelial cells the LDH release is relatively low.^15,29^ We were able to strongly increase the LDH release from the epithelial cells by replacing the tissue culture medium with DPBS during the incubation with *C. jejuni* (Fig. S3). We noticed that calcium excess in the culture medium reduces the *C. jejuni* cytotoxicity as has been noted for *C. coli* PldA.^30^

What is causing lysoPE-induced cell damage? It has been shown that the incorporation of even a small amount (1 mol.%) of fatty acids or lysolipids in lipid membranes creates instabilities in the lipid bilayer.^31^ One theory for LPL-induced cell damage is that LPLs, such as lysoPC, can evoke an oxidant stress-dependent transient membrane permeabilization in cells.^32^ Our results support this hypothesis as two antioxidants, vitamin E and DPPD, protect the cells from the LPLs damage. Both inhibitors reduced the lysoPE 14:0 induced cytotoxicity and also inhibited the intracellular membrane staining (Figure 4d). The mechanism of toxicity of lysoPE 14:0 may thus resemble the effect of as lysoPC leading to a stress-dependent transient membrane permeabilization.^32^

In conclusion, we for the first time identified *C. jejuni* lysolipids, especially lysoPE, as cytotoxic factor. The toxic short-tailed lysoPE induces hemolysis and induces oxidant stress-dependent membrane leakage in epithelial cells. Bacterial lysoPE can thus be considered as a novel virulence factor of *C. jejuni* and possibly other bacterial pathogens that generate large amounts of toxic lysoPE.

## Materials and methods

### Bacteria and mammalian cell culture

*C. jejuni* wildtype strain 81116, originally isolated from a human waterborne outbreak,^33^ its isogenic *pldA* mutant (*C. jejuni ΔpldA*), and the complemented *pldA* mutant (*C. jejuni ΔpldA +pldA*)^12^ were routinely grown on saponin agar as described.^12^ HeLa cells^34^ and Caco-2 cells (ATCC-HTB-37) were grown in 25 cm^2^ flasks in Dulbecco’s modified Eagle’s medium (DMEM) containing 10% fetal calf serum (FCS) at 37°C and 10% CO_2._

### Membrane isolation, lipid LC-MS/MS analysis, and extraction

*C. jejuni* membranes were collected by *N*-lauroylsarcosine assay,^35^ using sonication and Tris (PH 8.0) buffer instead of French pressure cell press and HEPES buffer, respectively. Lipid extraction and analysis were done as described before.^12^ For more information on membrane isolation and lipid extraction and analysis see supplementary materials (Supplementary Material 1). Commercial (lyso)phospholipids, lysoPE 14:0, lysoPE 16:0, lysoPE 18:0, lysoPG 14:0, lysoPG 18:0 and PE 16:0 were purchased from Avanti Polar Lipids Inc. (Alabama, USA) to investigate the effect of the length of LPL fatty acid tail on biological functions.

### Hemolysis and cytotoxicity assays

Hemolysis and cytotoxicity were determined as described^24^ using heat-treated (75°C, 30 min) bacteria,^36^ sonicated (3 × 60s) bacteria, isolated membranes, 10 μmol *C. jejuni* purified LPLs or 50 μmol commercial LPLs. Hemolysis was expressed as percentage of cell lysis (absorbance OD_420_) compared to the positive control (cells lysed with milliQ water). Host cell cytotoxicity was determined by measurement of the lactate dehydrogenase (LDH) release from 10^6^ tissue culture cells at 5 h after addition of *C. jejuni* at a bacteria to host cell ratio of 100:1, or of the indicated amount of LPL. When appropriate, host cells were pre-treated (16 h) with one of the antioxidants, vitamin E and *N,N’*-diphenyl-1,4-phenylenediamine (DPPD) (Sigma-Aldrich).^32^ For more detailed information on hemolysis and cytotoxicity assays see Supplementary Material 1. Data are expressed as the mean ±SEM of at least three independent experiments. Statistical significance was determined using two-way ANOVA analysis with Geisser-Greenhouse correction using Prism software (GraphPad, San Diego, CA).

### Confocal microscopy

Confocal microscopy^37^ was performed on cells (10^6^) incubated (5 h) with commercial lysoPE 14:0 or lysoPG 14:0 to visualize the lysoPE-induced cell damage. When appropriate antioxidants were added 16 h before LPL treatment and washed away before lysoPE exposure. Cells were fixed and membranes were stained with plasma membrane counterstain Wheat Germ Agglutinin (WGA) Alexa Fluor™ 488 Conjugate (W11261, Invitrogen). Nucleic acids were stained with DAPI (D21490, Invitrogen) without permeabilization. Images were collected on a Leica SPE-II confocal microscope.

## Supplementary Material

Supplemental Material

## Data Availability

The authors confirm that the data supporting the findings of this study are available within the article and its supplementary materials.
