# Supplementary material for: Campylobacter jejuni permeabilizes the host cell membrane by short chain lysophosphatidylethanolamines
Source: Gut Microbes. 2022 Jul 7;14(1):2091371. doi: 10.1080/19490976.2022.2091371 (PMC9272830; doi:10.1080/19490976.2022.2091371)
Supplement: Supplemental Material [file KGMI_A_2091371_SM3593.zip › Fig S1 S2 and S3.docx]

**Fig S1: A base peak chromatogram with MS-2 data (inlay) of the separated major phospholipid classes from wildtype *C. jejuni* strain 81116.** (A) lysoPE = lysophosphatidylethanolamine, (B) PE = phosphatidylethanolamine, (C) lysoPG = lysophosphatidylglycerol, (D) PG = phosphatidylglycerol.

**Fig S2: Cells membrane and nucleus staining.** Confocal microscopy of HeLa cells (10^6^ ) stained with WGA Alexa Fluor™ 488 Conjugate (green) to visualize the cell membrane and DAPI (blue) to stain the nuclei. HeLa cells incubated (A) with DPBS for 5 h, (B) with 500 µM lysoPE 14:0 for 5 h, (C) with 500 µM lysoPG 14:0 for 5 h, (D) pre-treated with 250 µM vitamin E for 24 h followed by incubation with 500 µM lysoPE 14:0. Red arrows point to nuclear membrane stained with WGA. White scale bars represent 5 μm.

**Fig S3**: **LDH release of Hela cells using DMEM or DPBS with and without the indicated *C. jejuni* strains.** Data shown are from three independent experiments with three different preparations of bacterial samples and presented as mean values ± standard deviation, **P < 0.01, ***P < 0.001, ****P < 0.0001, ns P > 0.1.
