# Supplementary material for: Campylobacter jejuni permeabilizes the host cell membrane by short chain lysophosphatidylethanolamines
Source: Gut Microbes. 2022 Jul 7;14(1):2091371. doi: 10.1080/19490976.2022.2091371 (PMC9272830; doi:10.1080/19490976.2022.2091371)
Supplement: Supplemental Material [file KGMI_A_2091371_SM3593.zip › Supplemental materials 1.docx]

**Supplementary Materials 1**

**Membrane isolation**

*C. jejuni* membranes were collected by *N*-lauroylsarcosine assay ^1^. In short, bacteria were grown in 100 ml HI medium for 16 h under microaerophilic conditions at 42°C. Bacterial pellets were collected by centrifugation (3,000×g, 10 min) and resuspended in 1 ml of 20 mM Tris-HCl, 5 mM EDTA, 20% sucrose, 0.15 mg lysozyme, pH 8.0 and incubated on ice for 40 min. Next, 40 μl of 0.5 M MgCl_2_ was added to the sample, which was then centrifugated (9,600×g, 20 min) to collect the supernatant containing the periplasm fraction. The remaining pellet was resuspended in 2 ml of ice cold 10 mM Tris-HCl, pH 8.0. Next, the cells were lysed by sonication for 3×60 second on ice. After centrifugation (6,200×g, 10 min) the membrane containing supernatant was collected. Finally, bacterial whole membranes were collected by ultracentrifugation at 100,000 g for 1 h at 4°C. The pellet was washed with 10 mM Tris-HCl, pH 8.0 and resuspended in 1 ml DPBS.

**Lipid LC-MS/MS analysis and extraction**

Lipid extraction and analysis were done as described before ^2^. In brief, bacteria were grown in 5 ml HI medium for 16 h under microaerophilic conditions at 42°C. The pellets were washed once with Dulbecco’s Phosphate Buffered Saline (DPBS) and resuspended in 0.8 ml DPBS and mixed with 3 ml chloroform/methanol (1:1 v/v). Supernatants were collected by centrifugation for 10 min at 4,600 rpm and mixed with 3 ml Milli-Q water. Phospholipids were collected and separated using a HILIC column (Kinetex, 2.6 μm) to resolve different phospholipid classes.

**Hemolysis assays**

*Campylobacter* cultures (5ml) were shaken (160 rpm) in T25 cell culture tested flasks (10790113, Corning) for 16 h at 37°C under microaerophilic conditions. The cultures were pelleted and resuspended in DPBS to OD_550_ of 1. Depending on the experimental design, resuspended bacteria were untreated, heat-treated for 30 min at 75°C, or sonicated for 3×60 second (VC-40, Sonics & Materials Inc, Newtown, USA). The bacterial culture, bacterial membranes or commercial (lyso) phospholipids (1 ml) were mixed with 0.25 ml of a 5% (v/v) horse, sheep, human or chicken erythrocyte suspension (Biotrading) in DPBS in a 1.5 ml Eppendorf tube. When live bacteria were used the caps of the Eppendorf tubes were perforated to allow gas exchange. After 16 h of incubation at 37°C under microaerophilic conditions, the bacteria and cells were resuspended and centrifuged (1,000×g, 5 min). The OD_420_ of the supernatants was measured as indicator of the degree of hemolysis. Negative (with 1 ml DPBS) and positive (with added 1 ml milliQ water) controls were included in all assays. Hemolysis was scored as percentage of cell lysis of the positive control. Data are expressed as the mean ±SEM of at least three independent experiments. Data were analyzed using Graphpad Prism software.

**Cytotoxicity assays**

The extent of membrane permeabilization was determined by measurement of the LDH release from the cells. Cytotoxicity toward tissue culture cells were determined using HeLa- and Caco-2 cells grown for 24 h in a 96-well plate in DMEM+10% FCS at 37°C in 10% CO_2_ atmosphere. The cells were washed with DPBS and the medium was replaced by 50 μl of DMEM (without FCS) or DPBS. *C. jejuni* strains were grown 16 h in HI broth, collected by centrifugation (3,000×g, 10 min), resuspended in DPBS, and added to the host cells at a bacteria to host cell ratio of 100:1. Ten μmol of lysoPE extracted from *C. jejuni* or 50 μmol of commercial lysoPE 14:0, lysoPE 16:0, lysoPE 18:0 or lysoPG 14:0 was added to 10^6^ tissue culture cells and kept for 5 h at 37°C in 10% CO_2_ before total cellular and secreted LDH from the cells were determined with the Cytotoxicity Detection Kit^PLUS^ (LDH) according to the manufacturer's protocol. Antioxidants vitamin E and *N*,*N*’-diphenyl-1,4-phenylenediamine (DPPD) purchased from Sigma-Aldrich (St. Louis, MO) were used to investigate whether they could inhibit the LPLs cytotoxicity. Hereto tissue culture cells were treated with one of the antioxidants for 16 h. Prior to the addition of the LPLs, the cells were washed with DPBS to eliminate noncellular interactions between the antioxidants and LPLs. The percentage of cytotoxicity after 5 h incubation was calculated as the percentage of LDH release compared to the total LDH concentration (% of cytotoxicity= 100*(LDH released/total LDH)). Presented results are from three individual assays performed in triplicate. Data were analyzed using Graphpad Prism software.

References

1. Hobb RI, Fields JA, Burns CM, Thompson SA. Evaluation of procedures for outer membrane isolation from *Campylobacter jejuni*. Microbiology (Reading) 2009; 155:979-988. doi: 10.1099/mic.0.024539-0 [doi].

2. Cao X, Brouwers J, van Dijk L, van de Lest C, Parker C, Huynh S, van Putten J, Kelly DJ, Wosten MM. The Unique Phospholipidome of the Enteric Pathogen Campylobacter jejuni: Lysophosholipids Are Required for Motility at Low Oxygen Availability. J Mol Biol 2020; . doi: S0022-2836(20)30459-9 [pii].
