# Supplementary figures and images for: Campylobacter jejuni permeabilizes the host cell membrane by short chain lysophosphatidylethanolamines
Source: Gut Microbes. 2022 Jul 7;14(1):2091371. doi: 10.1080/19490976.2022.2091371 (PMC9272830; doi:10.1080/19490976.2022.2091371)

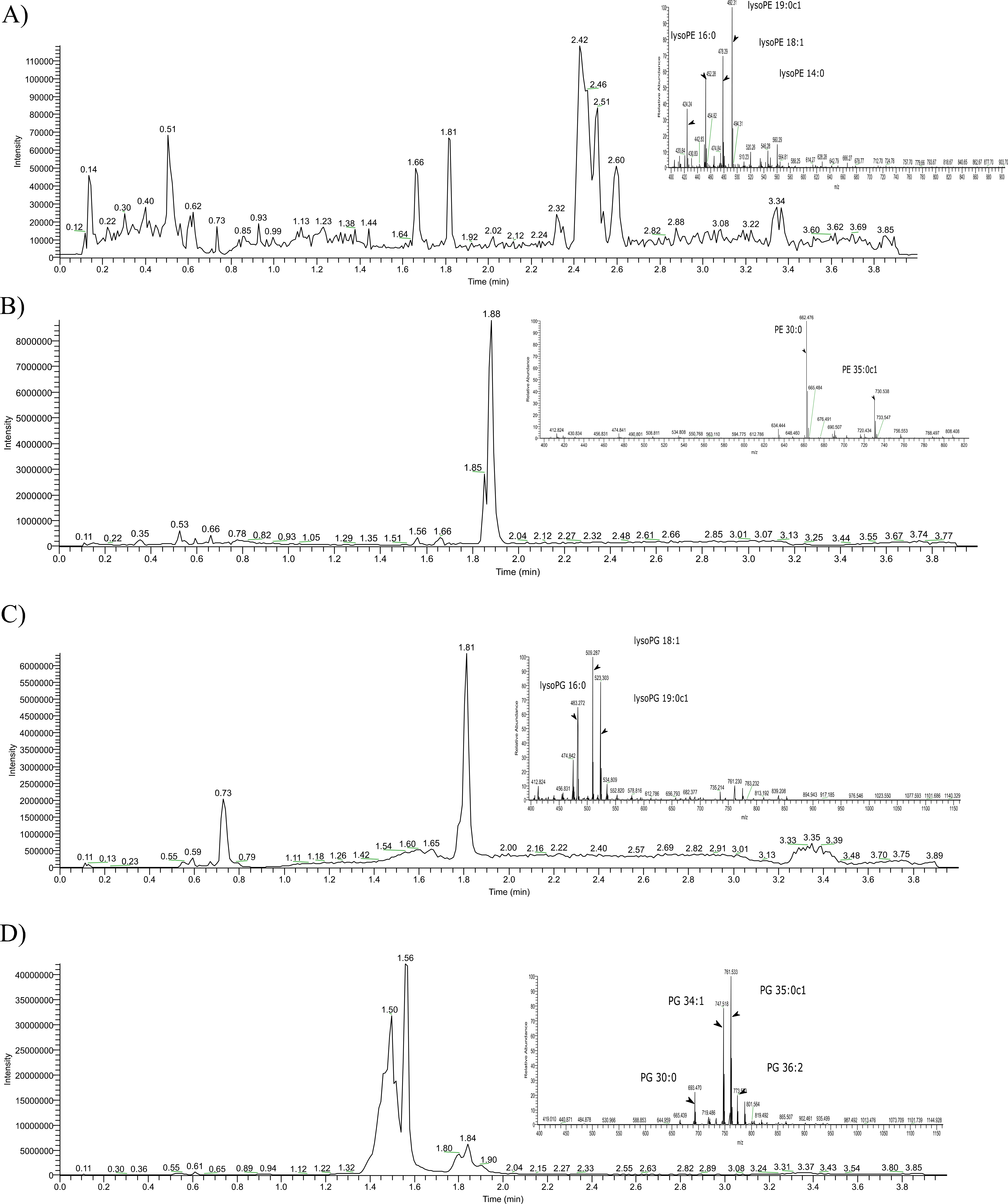

Supplement: Supplemental Material [file KGMI_A_2091371_SM3593.zip › Fig S1.jpg]

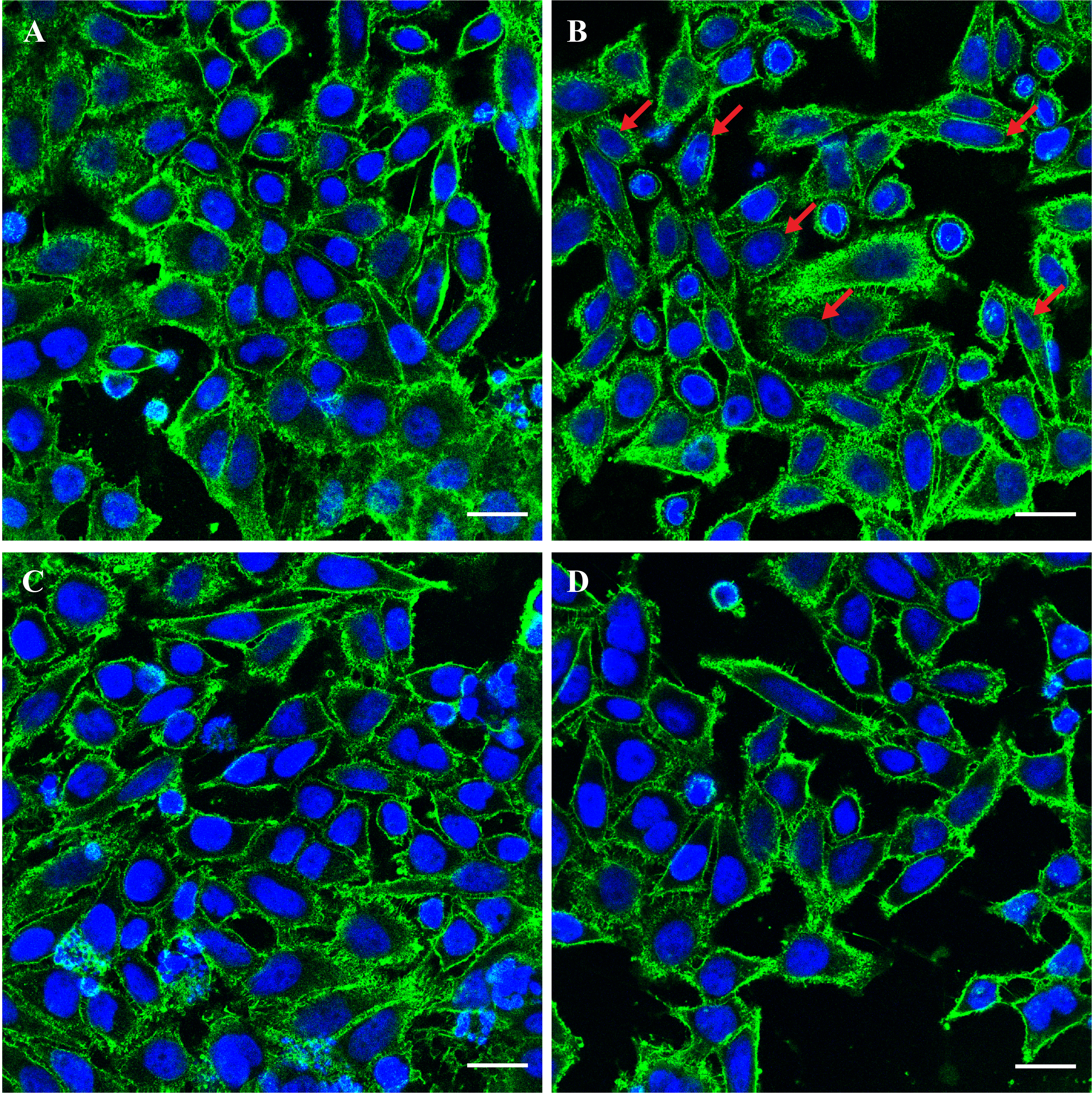

Supplement: Supplemental Material [file KGMI_A_2091371_SM3593.zip › Fig S2.tif]

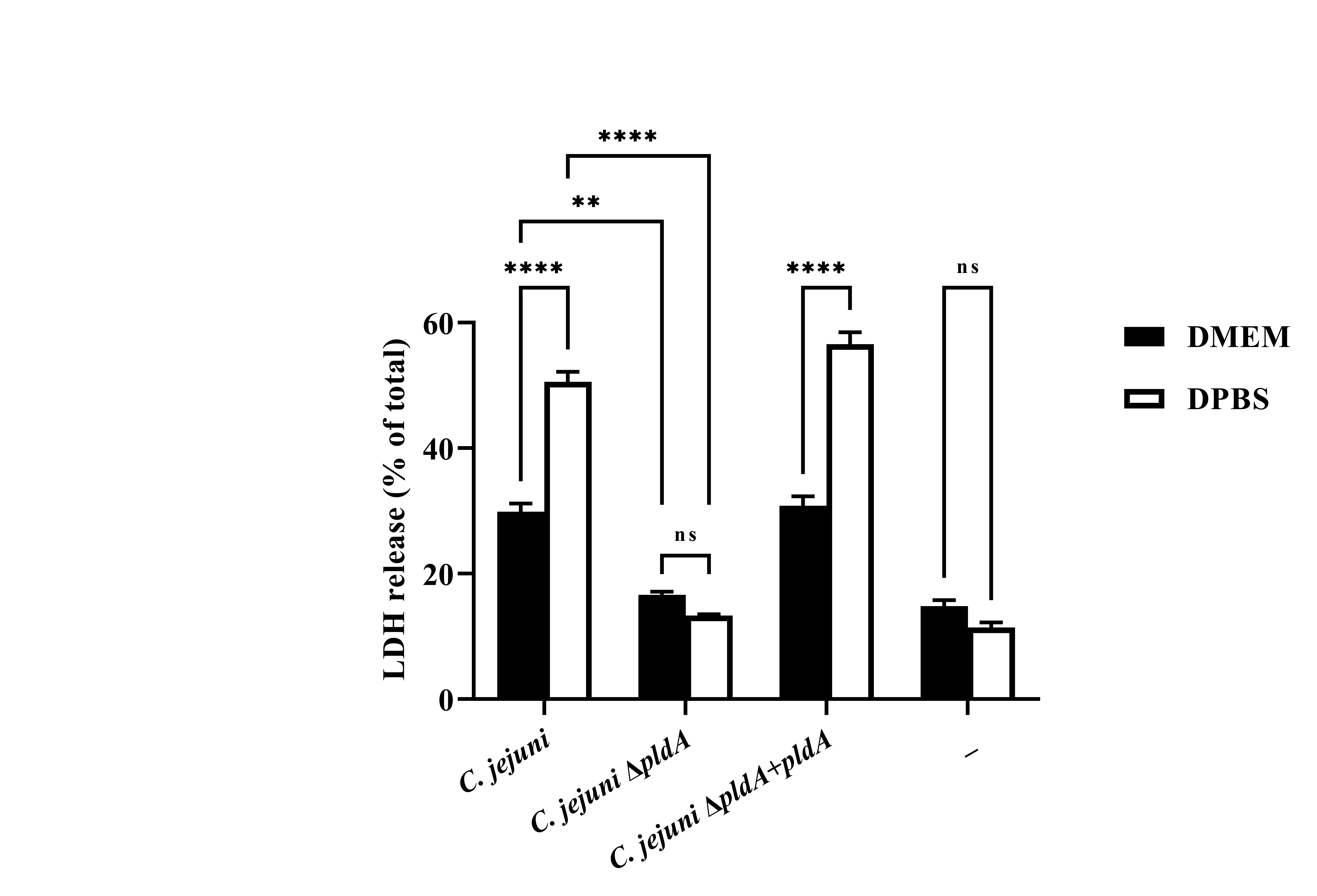

Supplement: Supplemental Material [file KGMI_A_2091371_SM3593.zip › Fig S3.tif]
